# A Bi_2_Te_3_-Filled Nickel Foam Film with Exceptional Flexibility and Thermoelectric Performance

**DOI:** 10.3390/nano12101693

**Published:** 2022-05-16

**Authors:** Taifeng Shi, Mengran Chen, Zhenguo Liu, Qingfeng Song, Yixiang Ou, Haoqi Wang, Jia Liang, Qihao Zhang, Zhendong Mao, Zhiwen Wang, Jingyvan Zheng, Qingchen Han, Kafil M. Razeeb, Peng-an Zong

**Affiliations:** 1 College of Materials Science and Engineering, Nanjing Tech University, Nanjing 210009, China; 202061103087@njtech.edu.cn (T.S.); chenmengran@njtech.edu.cn (M.C.); 202061203239@njtech.edu.cn (Z.M.); zwwang@njtech.edu.cn (Z.W.); 202161103082@njtech.edu.cn (J.Z.); 202161203210@njtech.edu.cn (Q.H.); 2 Key Laboratory of Flexible Electronics of Zhejiang Province, Ningbo Institute of Northwestern Polytechnical University, Ningbo 315103, China; iamzgliu@nwpu.edu.cn; 3 State Key Laboratory of High-Performance Ceramics and Superfine Microstructures, Shanghai Institute of Ceramics, Chinese Academy of Sciences, Shanghai 200050, China; qfsong@mail.sic.ac.cn; 4 Radiation Technology Institute, Beijing Academy of Science and Technology, Beijing 100875, China; ouyx16@tsinghua.org.cn; 5 Key Laboratory of Beam Technology of Ministry of Education, College of Nuclear Science and Technology, Beijing Normal University, Beijing 100875, China; wanghq@bnu.edu.cn; 6 State Key Laboratory of New Ceramics and Fine Processing, School of Materials Science and Engineering, Tsinghua University, Beijing 100084, China; liangj18@mails.tsinghua.edu.cn; 7 Institute for Metallic Materials, Leibniz Institute for Solid State and Materials Research, 01069 Dresden, Germany; q.zhang@ifw-dresden.de; 8 Micro-Nano Systems Centre, Tyndall National Institute, University College Cork, Dyke Parade, Lee Maltings, T12 R5CP Cork, Ireland; kafil.mahmood@tyndall.ie

**Keywords:** Bi_2_Te_3_, nickel foam, solvothermal method, thermoelectric film, flexible, TEG

## Abstract

The past decades have witnessed surging demand for wearable electronics, for which thermoelectrics (TEs) are considered a promising self-charging technology, as they are capable of converting skin heat into electricity directly. Bi_2_Te_3_ is the most-used TE material at room temperature, due to a high zT of ~1. However, it is different to integrate Bi_2_Te_3_ for wearable TEs owing to its intrinsic rigidity. Bi_2_Te_3_ could be flexible when made thin enough, but this implies a small electrical and thermal load, thus severely restricting the power output. Herein, we developed a Bi_2_Te_3_/nickel foam (NiFoam) composite film through solvothermal deposition of Bi_2_Te_3_ nanoplates into porous NiFoam. Due to the mesh structure and ductility of Ni Foam, the film, with a thickness of 160 μm, exhibited a high figure of merit for flexibility, 0.016, connoting higher output. Moreover, the film also revealed a high tensile strength of 12.7 ± 0.04 MPa and a maximum elongation rate of 28.8%. In addition, due to the film’s high electrical conductivity and enhanced Seebeck coefficient, an outstanding power factor of 850 μW m^−1^ K^−2^ was achieved, which is among the highest ever reported. A module fabricated with five such n-type legs integrated electrically in series and thermally in parallel showed an output power of 22.8 nW at a temperature gap of 30 K. This work offered a cost-effective avenue for making highly flexible TE films for power supply of wearable electronics by intercalating TE nanoplates into porous and meshed-structure materials.

## 1. Introduction

Flexible electronics (FEs) are an emerging electronic technology that integrates different material systems and different functional components on flexible substrates to form flexible devices and systems that can be stretched and bent. FEs subversively change the rigid physical form of traditional electronic devices and systems. So far, most FEs adopted traditional energy sources that require disassembly and cyclic charge/discharge, which bring in cost increases and inconvenience. For devices inserted into the human body, it is even hazardous. Thus, it is urgent to develop self-charging technology that could harvest energy from the ambient environment [1,2,3]. Flexible thermoelectrics (FTEs) could directly convert heat into electricity, and thus can be regarded as an alternative for powering FTEs that is long-lasting, reliable and safe [4]. The performance of thermoelectric (TE) materials can be evaluated via the TE figure of merit, zT = *S*^2^*σ*T/κ, where *S* represents the Seebeck coefficient, *σ* is the electrical conductivity, κ is the thermal conductivity, T represents the absolute temperature of the environment, and the term *S*^2^*σ* is defined as the power factor, *PF* [5]. To date, Bi_2_Te_3_, with a rhombohedral structure and a band gap of 0.15 eV, is the most widely used TE material, since it has the best TE performance at room temperature among all materials. Because the Bi and Te atoms are bonded via covalent and ionic bonding, the Bi_2_Te_3_ lattice is rigid and poor in flexibility [6,7]. Making Bi_2_Te_3_ flexible remains a present issue.

Most solid materials, including intrinsically brittle ones such as ceramic and glasses, can become flexible when their thickness is small enough. Taking the inflexible and rigid crystalline silicon as an example, when its thickness is as low as 100 nm, it becomes flexible and can be utilized in wearable electronics [8]. Hence, it is important to take thickness into account when measuring flexibility. A common way to investigate the flexibility of a material is to conduct a bending test by attaching the material to a regular cylinder with a series of reduced bending curvatures until breakage [9]. Recently, Peng et al. [10] put forward a figure of merit for flexibility, *f*_FOM_, which could be expressed by the yield strain, *ε*, i.e., the degree of elastic deformation preceding plastic deformation at a given thickness. The maximum *ε*, considered as the relative degree of elongation on the inner/outer surface, can be simply figured out from the geometry: *ε = h/2r*, where *h* represents the thickness and *r* represents the critical bending curvature prior to breakage. Bi_2_Te_3_, as a rigid material, is nonexclusively flexible if sufficiently thin. For example, Shang et al. [11] physically sputtered a thin layer of Bi_2_Te_3_ with a thickness of 0.75 μm on polyimide, and Na et al. [12] electro-chemically deposited a thin layer of Bi_2_Te_3_ with a thickness of 2.6 μm on stainless-steel sheet. The Bi_2_Te_3_ layer was then peeled off by attaching to a piece of epoxy tape. Their *f*_FOM_ values were lower than 4 × 10^−5^, which was ascribed to a small *h* but a large *r*. Polymers are intrinsically flexible, which can be used when forming composites with Bi_2_Te_3_. The Bi_2_Te_3_/PEDOT and Bi_2_Te_3_/PEDOT:PSS films synthesized by Wang [13] and Goo et al. [14], respectively, exhibited an increased *f*_FOM_ of an order of 1 × 10^−4^. Bi_2_Te_3_/cellulose fibers and Bi_2_Te_3_/rGO film synthesized by Jin [15] and Ding et al. [16] revealed a further increased *f*_FOM_ on the order of 4 × 10^−3^. Despite tremendous efforts to endow Bi_2_Te_3_-based thin films with a certain degree of flexibility, their thickness values are still small, implying restricted electrical and thermal loads, therefore limiting the maximal power output. Thus, attaining thicker and more flexible Bi_2_Te_3_ based film, meaning a larger *f*_FOM_, is a matter of urgency for real energy harvesting in wearable electronics.

Metal foams with porous and mesh structures have a great many properties that make them popular in a variety of applications, such as batteries and capacitors, among others [17,18]. Specifically, the capacity of metal foams to tolerate large strains (up to 60%) at a nearly constant stress can enable tremendous energy absorption without generating damaging peak stress, thus making them excellent candidates for energy absorption applications [17]. The energy from outer forces is also readily absorbed during bending deformation. Therefore, metal foams with porous and mesh structures are considered superior in terms of flexibility [19,20]. Nickel foam (NiFoam) is commercially available and cheap, and it has a high *σ* and an *S* with an absolute value second only to bismuth among all metals with negative *S* [21]. Open-cell NiFoam with a 3D thorough porous structure can allow Bi_2_Te_3_ nanoplates to enter, thus making a NiFoam/Bi_2_Te_3_ all-inorganic composite. It is expected to endow flexibility as well as to boost TE performance in the composite. In this work, we prepared a NiFoam/Bi_2_Te_3_ all-inorganic composite film using a solvothermal deposition method. The microstructures, performance and underlying mechanisms are discussed in detail.

## 2. Materials and Methods 

**Figure 1:**
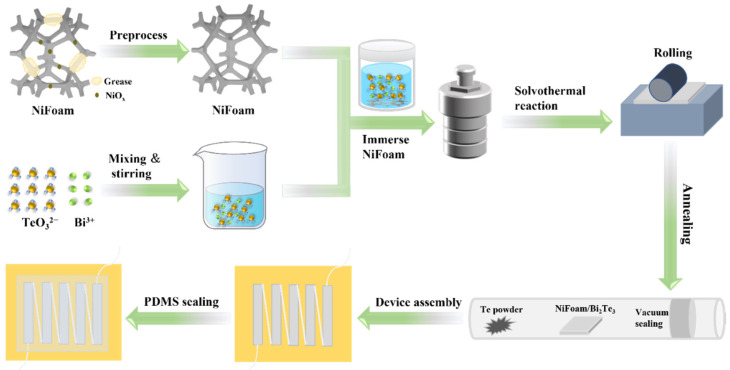
Schematic diagram of the synthesis of NiFoam/Bi_2_Te_3_ composite film and module fabrication.

Material synthesis and module integration: The material preparation and device synthesis process are shown in Figure 1. Firstly, a 20 mm × 20 mm segment of NiFoam was cut out and added into 3 M HCl aqueous solution under ultrasonication for 10 min. Then, the NiFoam was washed with deionized water and ethanol under ultrasonication for 10 min. This pretreatment could remove the oxide layer, as well as other contaminants such as grease. After that, the NiFoam was vacuum-dried at 303 K for 3 h. Secondly, 1 mM bismuth nitrate pentahydrate (Bi (NO_3_)_3_·5H_2_O, ≥99%, Aladdin, Los Angeles, USA), 1.5 mM sodium tellurite (Na_2_TeO_3_, ≥97%, Meryer, Shanghai, CHN), and 0.23 g polyvinylpyrrolidone (PVP, K30, Rhawn, Shanghai, CHN) were added into 30 mL ethylene glycol (EG, ≥99%, Yonghua Chemical, Suzhou, CHN) under stirring for 10 min. Then, 0.4 g sodium hydroxide (NaOH, ≥99%, Xilong Scientific, Shantou, CHN) was added under stirring until the precursor solution was clear. The precursor solution was then added along with the NiFoam into an autoclave, which was heated at 453 K for 12 h. The result was vacuum-dried at 333 K for 6 h to obtain the NiFoam/Bi_2_Te_3_ composite. In order to suppress the volatilization of Te in the high temperature film, the NiFoam/Bi_2_Te_3_ composite was pressed under 1 MPa and sealed together with Te powder in a quartz tube in vacuum. Then, the quartz tube was heated at 573 K for 90 min, and allowed to cool down to room temperature naturally. The obtained NiFoam/Bi_2_Te_3_ film was cut into legs with sizes of approximately 4 mm × 17 mm × 0.16 mm. Five such legs were integrated thermally in parallel and electrically in series with silver paste on the polyimide tape. In order to prevent possible oxidation, all the legs were sealed with polydimethylsiloxane (PDMS).

Microstructure characterization and performance measurement: The phases were characterized using X-ray diffractometry (XRD, Rigaku, Ultima IV) via CuKα radiation (1.5418 Å). GAGS software was used to perform Rietveld refinement, with an R_wp_ of approximately 5%, on the Bi_2_Te_3_ powder made by the solvothermal method and the annealed NiFoam/Bi_2_Te_3_ composite film. The microstructure of the film was characterized using field-emission scanning electron microscopy (FE-SEM, ZEISS, MERLIN Compact). A transmission electron microscope (TEM, Jeol, JEM 2010) was used to observe the microstructures of the nanoplates. XPS measurement was conducted on a Thermo Scientific ESCALAB 250Xi with a monochromated aluminum X-ray source (hv = 1486.6 eV), at a beam energy of 15 kV, a beam current of 10 mA and an incident angle of 58°, while an electron flood gun was used for charge compensation. The C peak at 284.6 eV was used to model other peaks and the Shirley algorithm was utilized to deduct base lines. The four-point probe system (LRIPER, LPPS100A) was used to test the electrical conductivity via the Van der Pauw method. The Seebeck coefficient was measured using a home-made system reported elsewhere [22]. Flexibility was characterized by attaching the film to the outer surfaces of glass tubes with different radii and evaluating the rate of change in resistivity. Tensile strength testing was performed on a sample with a size of 10 mm × 12 mm × 0.17 mm using a commercial multifunctional mechanical characterization device (EAST SUN, DR-5010A).

## 3. Results and Discussion

### 3.1. Phases and Microstructures

**Figure 2:**
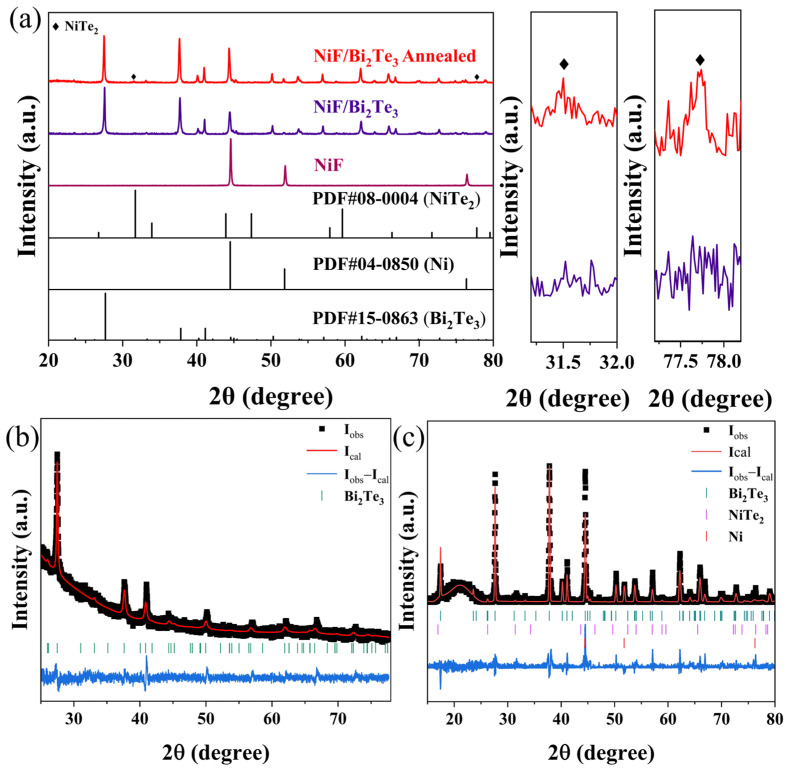
(**a**) XRD patterns of NiFoam and NiFoam/Bi_2_Te_3_ composite film before and after annealing. Rietveld refinement results of (**b**) solvothermally prepared Bi_2_Te_3_ powder and (**c**) annealed NiFoam/Bi_2_Te_3_ composite.

**Figure 3:**
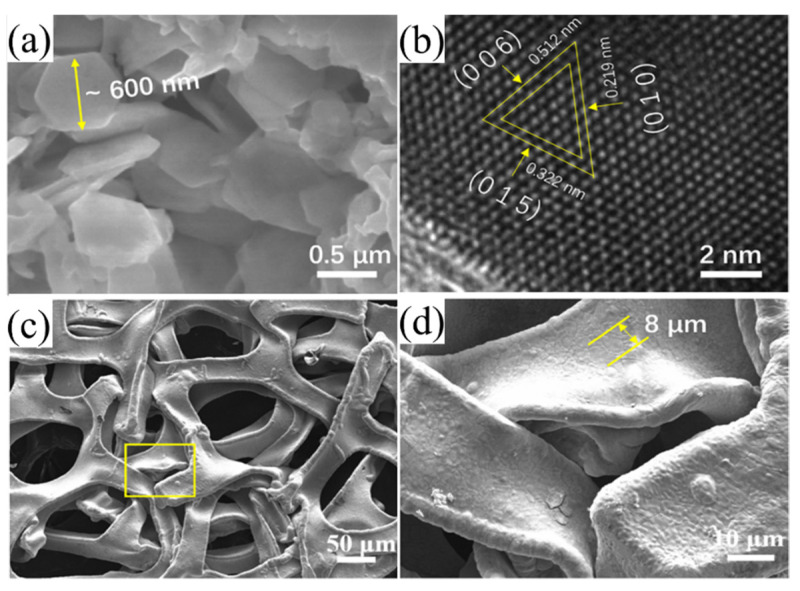
(**a**) SEM and (**b**) TEM images of the solvothermally deposited Bi_2_Te_3_ nanoplates; (**c**) low- and (**d**) high-magnification SEM images of NiFoam.

Figure 2a shows the XRD patterns of the NiFoam and the NiFoam/Bi_2_Te_3_ film before and after annealing. The XRD patterns of the pretreated NiFoam were consistent with the standard peaks of Ni (PDF#04-0850), and no other impurity phases were observed. It was considered that the possible oxide layers and grease on the surface of the NiFoam skeletons were removed during the pretreatment [23]. The raw materials Bi (NO_3_)_3_·5H_2_O and Na_2_TeO_3_ were used as Bi and Te sources, respectively. EG could coherently act as solvent and reducing agent, as the hydroxyl groups in EG are reducible under alkaline conditions with NaOH. Bi (NO_3_)_3_·5H_2_O and Na_2_TeO_3_ were first reduced to Bi and Te, respectively, and then Bi_2_Te_3_ was produced via atomic bonding. PVP could reduce the activities of Bi and Te for slow and stable generation of Bi_2_Te_3_ [24]. As shown in Figure 3a, the Bi_2_Te_3_ nanoplates revealed sharp edges and a regular hexagonal shape with an average diameter of approximately 600 nm, consistent with the TEM investigations. The crystal spacings of 0.219, 0.322 and 0.512 nm were assigned to the (0 1 0), (0 1 5) and (0 0 6) faces, respectively, of Bi_2_Te_3_ (Figure 3b).

The XRD pattern of the NiFoam/Bi_2_Te_3_ film prepared via the solvothermal method revealed both Ni peaks (PDF#04-0850) and Bi_2_Te_3_ peaks (PDF#15-0863), indicating that Bi_2_Te_3_ was in situ deposited into NiFoam (Figure 2a). After annealing, minor diffraction peaks of NiTe_2_ (PDF#08-0004) were also found as marked by the rhombi, indicating possible formation of a NiTe_2_ transition layer at the interface of Ni and Bi_2_Te_3_ during annealing [25]. This was confirmed by later XPS measurement. The relative intensity of the diffraction peak (1 0 1 0) at 37.8° before and after annealing was much greater than that of the Bi_2_Te_3_ standard card. This could be attributed to the pressing process, which possibly caused optimal orientation. As shown in the Rietveld refinement results in Figure 2b,c, the exact stoichiometry of the Bi_2_Te_3_ powder made using the solvothermal method was Bi_1_._76_Te_3_._24_. After depositing into NiFoam and subsequent annealing, the stoichiometry was refined to Bi_1_._9_Te_3_._12_, which was regarded as due to the formation of minor amounts of NiTe_2_ with a mass ratio of 3%. The weight fractions of Bi_1_._9_Te_3_._12_ and Ni were 72% and 25%, respectively.

**Figure 4:**
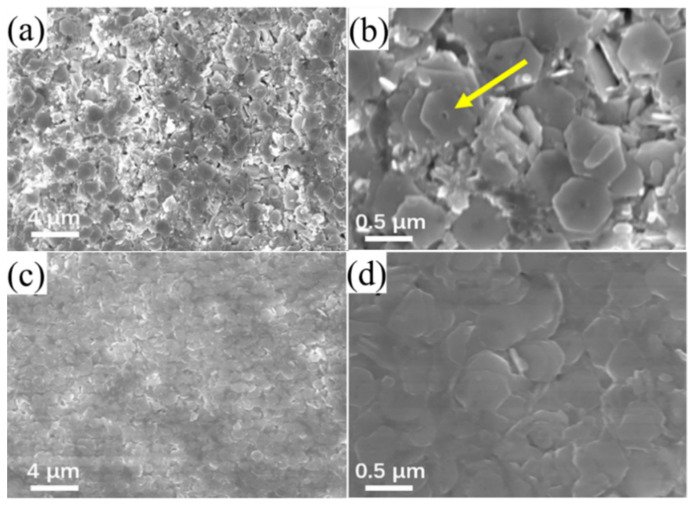
SEM images of NiFoam/Bi_2_Te_3_ composite film before (**a**,**b**) and after annealing (**c**,**d**) with low to high magnifications.

Figure 3c shows a low-magnification image of the NiFoam where the three-dimensional porous structure is visible. The diameter of the through-holes was ~200 μm. The surface of the nickel skeleton was smooth after cleaning, with a skeleton width of ~50 μm (Figure 3d). The interior grain boundaries were clear after HCl corrosion. The Ni grain size was ~8 μm as indicated. As shown in Figure 4a,b, Bi_2_Te_3_ was successfully solvothermally deposited onto/into the NiFoam. The three-dimensional through-holes of the NiFoam were completely filled with Bi_2_Te_3_ nanoplates, and the Ni skeleton was also completely covered. The interfaces between the nanoplates were clear, implying that the interfacial bonding between them was weak. The black circle in the center of the nanoplates (Figure 4b) was reported to be due to Ostwald ripening at low reaction temperatures (≤463 K) [26]. After annealing, the film (Figure 4c,d) became relatively dense and smooth. Annealing improved the atomic diffusing and blurred the interfaces between the nanosheets, which was conducive to carrier transport [27]. This is corroborated via the improvement in the *σ* and mechanical stability of the films after annealing.

**Figure 5:**
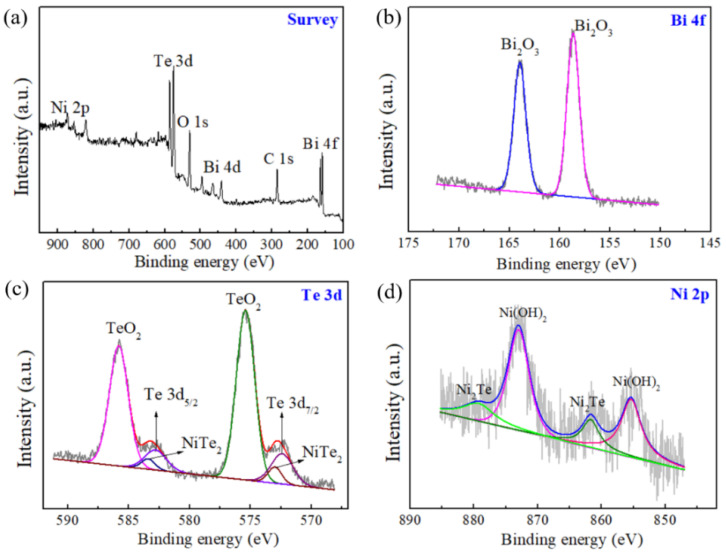
X-ray photoelectron spectra of (**a**) survey scan, (**b**) Bi 4f region, (**c**) Te 3d region, and (**d**) Ni 2p region for the annealed NiFoam/Bi_2_Te_3_ film.

XPS spectra of the annealed NiFoam/Bi_2_Te_3_ film are shown in Figure 5. The survey scan in Figure 5a showed the major Bi, Te, and Ni peaks. There were some peaks of O and C. The presence of oxygen was mainly due to surface oxidation of Bi_2_Te_3_. The presence of carbon was due to surface hydrocarbon contaminants. In the Bi 4f curve (Figure 5b), two peaks at ≈158.7 and 163.9 eV were attributed to Bi 4f_7/2_ and Bi 4f_5/2_, respectively, revealing the existence of Bi_2_O_3_ and corresponding well to the reference values [28]. From the Te 3d curve (Figure 5c), the binding energies of Te 3d_5/2_ and Te 3d_3/2_ were 572.4 and 582.8 eV, respectively, which also corresponded well to the reference values, while the two peaks at 573 and 583.4 eV were attributed to NiTe_2_ [29]. The other two strong peaks were attributed to TeO_2_, confirming the surface oxidation of Bi_2_Te_3_. In the Ni 2p curve (Figure 5d), the peaks at 873.70 and 855.5 eV were attributed to Ni(OH)_2_, according to the NIST XPS database [30], which might be due to a surface reaction between Ni and NaOH.

### 3.2. Mechanical Properties

**Figure 6:**
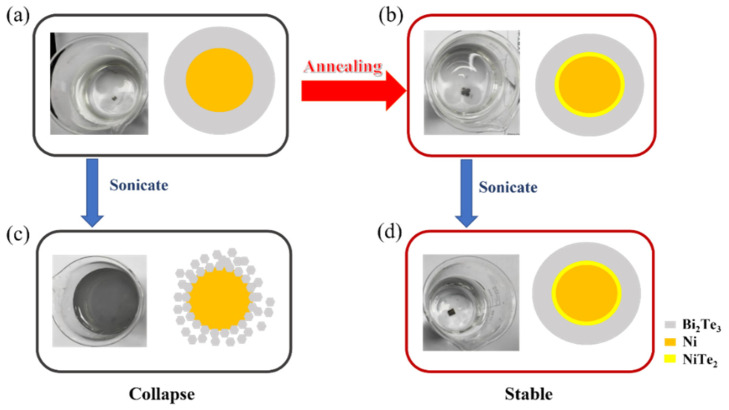
Schematic diagrams of the changes in the NiFoam/Bi_2_Te_3_ interface (**a**) before and (**b**) after annealing, and photos showing possible stripping under sonication for NiFoam/Bi_2_Te_3_ film (**c**) before (collapse) and (**d**) after annealing (stable).

Both XRD patterns and XPS spectra revealed the formation of the NiTe_2_ phase during annealing. The formed NiTe_2_ was sandwiched between Ni and Bi_2_Te_3_, forming a core–shell structure (Ni/NiTe_2_/Bi_2_Te_3-x_) (Figure 6b). This enhanced the mechanical bonding between Ni and Bi_2_Te_3_. Therefore, the mechanical stability and durability could be improved. Since nickel and Bi_2_Te_3_ were regarded as simply combined via the Van der Waals force before annealing (Figure 6a), Bi_2_Te_3_ could be easily stripped off from the nickel frame during ultrasonication as can be seen in Figure 6c. This did not occur for the film after annealing (Figure 6d).

**Figure 7:**
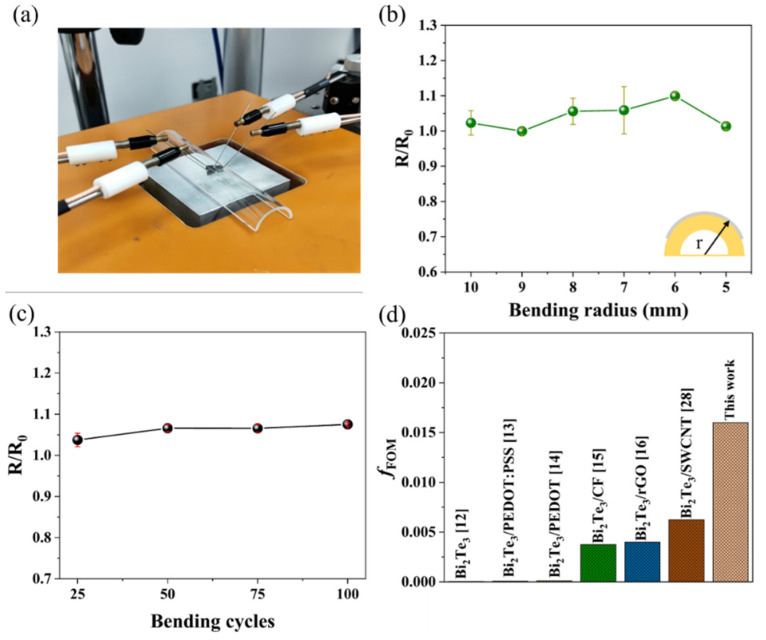
(**a**) Photograph illustrating resistivity test via the four-probe method where the NiFoam/Bi_2_Te_3_ film was bent and attached to a glass tube; (**b**) ratio of resistivity at bending state (R) to flat state (R_o_) at different bending radii (*r*); (**c**) R/R_o_ under a series of bending cycles at *r* = 5 mm; (**d**) the figure of merit for flexibility (*f*_FOM_) of Bi_2_Te_3_-based TE films reported and in this work.

Figure 7a is a photograph of a composite film undergoing flexibility testing. When the bending radius was reduced from 10 mm to 5 mm (Figure 7b), the decrease in *σ* was less than 10% compared to the flat state. The film was then subjected to 25–100 bending cycles at a bending radius of 5 mm. The decrease in *σ* was still less than 10% (Figure 7c). This indicates excellent mechanical flexibility of the NiFoam/Bi_2_Te_3_. Figure 7d lists the value of the *f*_FOM_ from recently published Bi_2_Te_3_-based alloys and composites, and it can be seen that our NiFoam/Bi_2_Te_3_ film had a *f*_FOM_ value of more than three times higher than others, which shows that the film has excellent flexibility.

**Figure 8:**
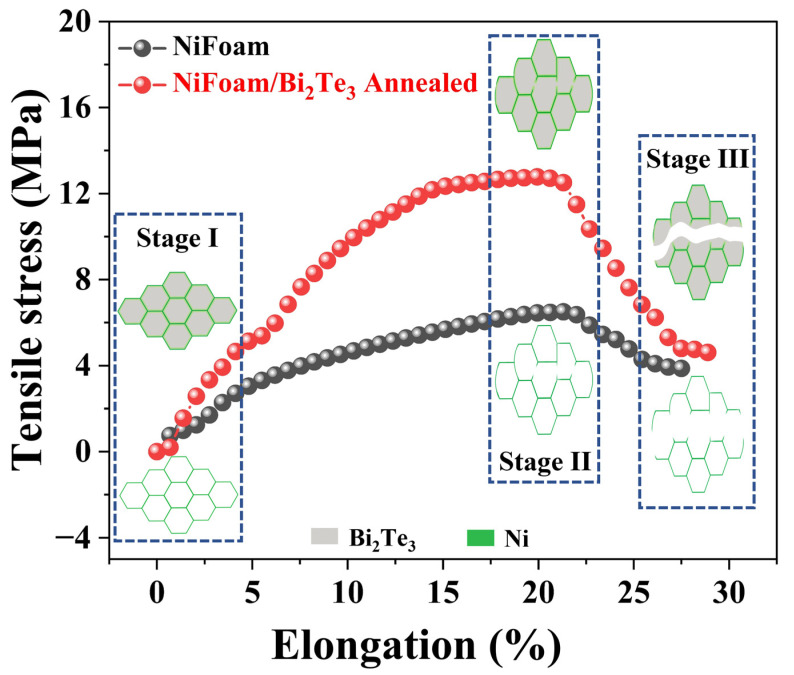
Curves of tensile strength vs. elongation of NiFoam and NiFoam/Bi_2_Te_3_ film; the inset is the schematic diagram of tensile processes of NiFoam and the annealed NiFoam/Bi_2_Te_3_ composite film.

As is known, wearable electronics are sometimes under tensile stress. It is thus significant to characterize the tensile strength of the TE films for wearable usage. Figure 8 shows the tensile–elongation curves of NiFoam and the annealed NiFoam/Bi_2_Te_3_ film. The unit shape of the NiFoam could be regarded as a regular hexagon (Figure 8, Stage I). When the hexagonal NiFoam began to stretch, it first deformed from a regular hexagon to a rectangle, and the theoretical elongation according to geometry at this time was ~17%. After that, there was considered to be an elastic deformation period and a yield stage, which were not obvious in the curve. Then, a plastic stage continued until the maximum stress, 6.5 ± 0.02 MPa. The elongation at this point was 21% (Figure 8, Stage II). After that, the instability fracture stage came, showing necking deformation until breakage. At this point, the maximum elongation was 27.5% (Figure 8, Stage III). For the NiFoam/Bi_2_Te_3_ film, the maximum tensile strength was 12.7 ± 0.04 MPa, which was twice that of NiFoam, due to the strengthening effect of the bonded Bi_2_Te_3_. As shown in Figure 8, during stage I to stage II, the Bi_2_Te_3_ was first compressed and stretched concurrently with the initial geometry deformation of NiFoam. Bi_2_Te_3_ has a rigid intrinsic nature; it is weaker in tension than it is in compression. The tensile strength is often at least one order of magnitude lower than the compressive strength for many brittle materials [31]. It was considered that horizontal cracks would form first in Bi_2_Te_3_ and then extend to the skeleton of the NiFoam. The maximum elongation, 28.8%, did not change much due to the rigid nature of Bi_2_Te_3_.

As shown in Table 1, compared with the TE films reported, the as-prepared NiFoam/Bi_2_Te_3_ film exhibited the best elongation overall. Its tensile stress of 12.7 ± 0.04 MPa was higher than that of CNT/PDMS, PPBH/CNT/PUBI, or PEDOT/SWCNT/BC, and comparable to that of DMSO/PEDOT:PSS, PEDOT:PSS/Rubber, or PVDF/Ni, which were based on intrinsic flexible organics. Moreover, as the NiFoam/Bi_2_Te_3_ film showed the best *PF*, which was ~2 orders of magnitude higher than the above materials, it can be concluded that the NiFoam/Bi_2_Te_3_ film displayed the best comprehensive performance for flexible energy harvesting.

### 3.3. Thermoelectric Properties and Power Generation of Module

**Figure 9:**
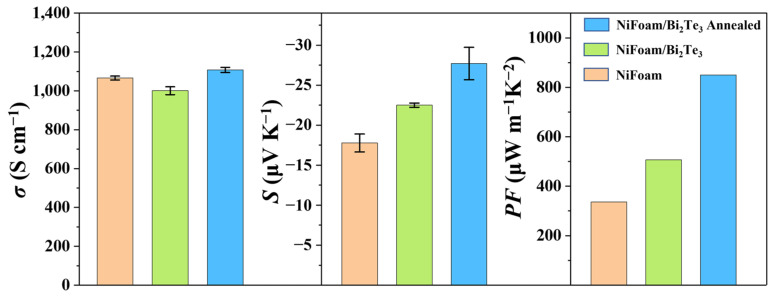
Electrical conductivity (*σ*), Seebeck coefficient (*S*) and power factor (*PF*) of NiFoam and NiFoam/Bi_2_Te_3_ film before and after annealing.

As shown in Figure 9, after solvothermal deposition of Bi_2_Te_3_ nanoplates onto/into the three-dimensional networked NiFoam, *σ* was slightly decreased, from 1066.1 S cm^−1^ to 1000.9 S cm^−1^. This was due to the much lower intrinsic *σ* in Bi_2_Te_3_ compared to NiFoam, as the mass fraction of Bi_2_Te_3_ reached 58.2%. However, filling Bi_2_Te_3_ into the through-holes of NiFoam could provide additional carrier transport paths, which might increase mobility. This would help maintain the *σ* without much degradation. After annealing, the *σ* was increased from 1000.9 S cm^−1^ to over 1107.8 S cm^−1^. Because annealing could reduce defects in the Bi_2_Te_3_, decrease the boundary density, and bridge the nickel skeleton and Bi_2_Te_3_ by generating transition phases, it was possible for it to increase the mobility. The absolute value of *S* of the NiFoam/Bi_2_Te_3_ film increased from 17.7 μV K^−1^ for NiFoam to 22.2 μV K^−1^, due to the weighting effect as the intrinsic *S* of Bi_2_Te_3_ was higher than NiFoam. After annealing, the absolute value of *S* was further increased to 27.7 μV K^−1^, which was due to the reduction in carrier concentration caused by NiTe_2_ formation and evaporation of trace Te. The *PF* of the composite film before annealing reached 493 μW m^−1^ K^−2^, which was 1.5 times that of pure NiFoam. After annealing, the *PF* further increased to 850 μW m^−1^ K^−2^, 2.5 times that of pure NiFoam. The geometry of the Bi_2_Te_3_ filling the foam pores could be approximated as being spherical, and therefore the thermal conductivity of the as-prepared NiFoam/Bi_2_Te_3_ film could be estimated to be 1.7 W m^−1^ K^−1^ based on the Woodside and Messmer model [38], $\kappa_{c}=\kappa_{m}^{p}\times \kappa_{s}^{1-p}$, where $\kappa_{c}$, $\kappa_{m}$ and $\kappa_{s}$ are the thermal conductivity of the composite, Bi_2_Te_3_, and nickel, respectively, and p is the original porosity. The corresponding TE figure of merit, zT, was thus calculated to be 0.15, which is lower than the magnetically sputtered Bi_2_Te_3_-based films [39]. Nevertheless, it had a much larger thickness (160 μm), two orders of magnitude higher, than the sputtered film, implying a much higher tolerance of electrical and thermal load and thus a higher power output.

**Figure 10:**
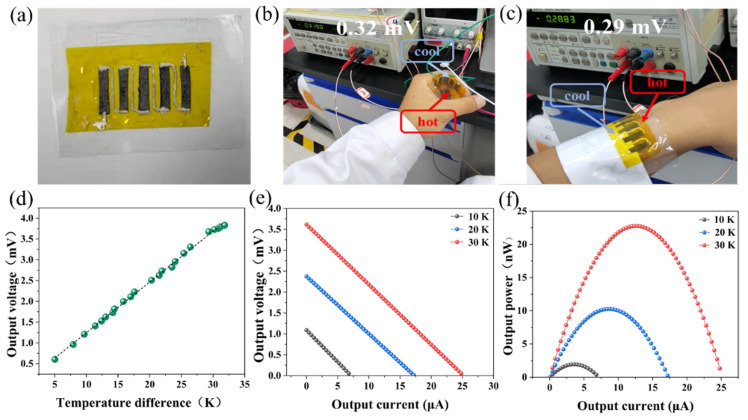
(**a**) Photograph of the assembled TE module; (**b**) voltage output when the TE was held against the wrist or (**c**) attached onto the arm with one end exposed to the air; (**d**) output voltage of the TE module at different temperature differences; (**e**) output voltage and (**f**) output power of the TE module under temperature differences of 10 K, 20 K and 30 K.

In order to demonstrate power generation by a TE module based on the as-prepared NiFoam/Bi_2_Te_3_ composite films, five strips with a size of~ 4 mm × 17 mm × 0.16 mm were cut out and connected electrically in series using silver paste on a piece of polyimide film (Figure 10a). As shown in Figure 10b, when the TE module was held with one end on the wrist with the other end exposed to air, the maximum open circuit voltage was 0.32 mV. When one end of the TE module was attached to arm skin, while a piece of cloth was placed between the other end and the skin for increasing thermal resistance (to develop a thermal gradient), a maximum open circuit voltage was of 0.29 mV was achieved (Figure 10c). Therefore, the TE module could be used to collect skin heat and convert it into power at ambient temperature. In order to further demonstrate power generation by the TE module under different temperature differences, one end of the TE module was attached to a heating plate and the other end was exposed to air. By adjusting the power of the heating plate, different temperature differences, ΔT, could be established (Figure 10d). The open circuit voltages of the TE module were 0.61 mV, 1.86 mV, 3.12 mV, and 3.75 mV at temperature differences of 5 K, 15 K, 25 K, and 30 K, respectively. These measured values were consistent with the theoretical values evaluated via V_0_ = ΔT × |*S*| × N, where |*S*| is the absolute *S* and N is the total number of thermoelectric legs. To demonstrate the power output of the TE module under various temperature differences, an external resistor was connected to form a power measurement circuit based on voltammetry. The power output *P* of the module could thus be expressed as:

(1) $$P=\frac{E^{2}}{\frac{{\left(R_{ex}-R_{in}\right)}^{2}}{R_{ex}}+4R_{in}}\;$$

where *R_ex_* is the resistance of the external resistor, *R_in_* is the total internal resistance of the module and the ammeter. For a fixed ΔT, the output voltage (*E*) is a constant, while the *P* could reach maximum at *R_ex_* = *R_in_*. Figure 10e,f shows the curves of power and voltage output vs. the current of the circuit at ∆T = 10 K, 20 K, and 30 K, respectively. At ∆T = 10 K, *R_ex_* = 150 Ω and *P*_max_ = 1.95 nW, with the voltage and the current being 1.09 mV and 6.87 mA, respectively. At ∆T = 20 K, *R_ex_* = 140 Ω and *P*_max_ = 10.28 nW, with the voltage and current being 2.38 mV and 11.24 mA, respectively. At ∆T = 30 K, *R_ex_* = 137 Ω and *P*_max_ = 22.76 nW, with the voltage and current being 3.62 mV and 24.92 mA, respectively. The TE module could provide an output power of several tens of nW under a small temperature difference. Moreover, it is flexible and bendable. Thus, our NiFoam/Bi_2_Te_3_ based thermoelectric generator has the potential to be used for a variety of miniature sensors and wearable devices, such as for powering temperature and humidity sensors, blood pressure sensors, heartbeat sensors, bracelets, etc.

## 4. Conclusions

In conclusion, we prepared a NiFoam/Bi_2_Te_3_ composite film that exhibited a high *PF* of 850 μW m^−1^ K^−2^ and excellent mechanical flexibility as well as high tensile strength. Its figure of merit for flexibility could reach 0.016, and its tensile strength and breaking elongation were 12.7 ± 0.04 MPa and 28.8%, respectively. This composite material was fabricated by solvothermal deposition of Bi_2_Te_3_ nanoplates into/onto porous NiFoam followed by annealing. The generation of a transition layer of NiTe_2_ during annealing could enhance bonding between the NiFoam skeleton and the Bi_2_Te_3_, thereby boosting its mechanical stability. A thermoelectric (TE) module was assembled with five legs with dimensions of ~4 mm × 17 mm × 0.16 mm. The output voltage of the TE module was 3.75 mV and the output power was 22.8 nW at a temperature difference of 30 K. Due to the low cost of commercial NiFoam as well as the simplicity of synthesis, the Bi_2_Te_3_/NiFoam film demonstrated great potential for powering wearable electronics by harvesting energy from human skin. This work provides a cost-effective method for making exceptionally flexible and high-performance TE films for energy harvesting by intercalating TE nanoplates into a porous meshed nickel structure that is intrinsically flexible.

## Figures and Tables

**Table 1:** Tensile strengths and power factors of TE films.

Composition	Tensile Strength (MPa)	Elongation (%)	Power Factor (μW m^−1^ K^−2^)	Ref.
CNT/PDMS Foam	0.78	20.6	2.9	[32]
DMSO/PEDOT:PSS	38.97	6.6	108.9	[33]
PEDOT:PSS/Rubber	20.12	4.0	19.1	[34]
PVDF/Ni nanowires	25.3	9.0	24.3	[35]
PPBH/CNT/PUBI	6.11	3.8	6.3	[36]
PEDOT/SWCNT/BC	1.6	2.1	12.0	[37]
Ni Foam/Bi_2_Te_3_	12.7 ± 0.04	28.8	850.0	This work

Notes: CNT: carbon nanotubes, PDMS: polydimethylsiloxane, DMSO: Dimethyl sulfoxide, PEDOT:PSS: Poly(3,4-ethylenedioxythiophene):poly(styrene sulfonate), PVDF: poly(1,1-difluoroethylene), PPBH: polymer particles bearing many small bumps and crosslinkable hydroxyl groups on their surfaces, PUBI: water dispersible polyurethane with blocked terminal isocyanate groups [36], PAA: Polyacrylic acid, CA: Cellulose acetate, SWCNT: single-wall carbon nanotube, BC: Bacterial cellulose.

## Data Availability

Not applicable.
